# Generation of glucagon‐like peptide‐2‐expressing *Saccharomyces cerevisiae* and its improvement of the intestinal health of weaned rats

**DOI:** 10.1111/1751-7915.12412

**Published:** 2016-09-19

**Authors:** Zhongwei Zhang, Xiaodong Wu, Lili Cao, Zhengdong Zhong, Yan Zhou

**Affiliations:** ^1^ Department of Intensive Care UnitWest China HospitalSichuan UniversityChengduSichuan610041China; ^2^ Hospital of Chengdu Office of People's Government of Tibetan Autonomous RegionChengduSichuan610041China; ^3^ Medical SchoolChengdu UniversityChengduSichuan610041China; ^4^ Sichuan Academy of Medical Sciences & Sichuan Provincial People's HospitalChengduSichuan610041China

## Abstract

We aimed to assess the feasibility of enhancing the intestinal development of weaned rats using glucagon‐like peptide‐2 (GLP‐2)‐expressing *Saccharomyces cerevisiae* (*S. cerevisiae*). GLP‐2‐expressing *S. cerevisiae* (GLP2‐SC) was generated using a recombinant approach. The diet of weaned rats was supplemented with the GLP2‐SC strain. The average daily gain (ADG), the intestinal morphology and the activities of the digestive enzymes in the jejunum were tested to assess the influence of the GLP2‐SC strain on intestinal development. The proliferation of rat enterocytes was also assessed in vitro. The study revealed that the ADG of the weaned rats that received GLP2‐SC was significantly greater than that of the controls fed a basal diet (Control) and *S. cerevisiae* harbouring an empty vector (EV‐SC) (*P* < 0.05) but was equivalent to that of positive control rats fed recombinant human GLP‐2 (rh‐GLP2) (*P* > 0.05). Furthermore, GLP2‐SC significantly increased villous height (*P* < 0.01) and digestive enzyme activity (*P* < 0.05) in the jejunum. Immunohistochemistry analysis further affirmed that enterocyte proliferation was stimulated in rats fed the GLP2‐SC strain, as indicated by the greater number of enterocytes stained with proliferative cell nuclear antigen (*P* < 0.05). In vitro, the proliferation of rat enterocytes was also stimulated by GLP‐2 expressed by the GLP2‐SC strain (*P* < 0.01). Herein, the combination of the GLP‐2 approach and probiotic delivery constitute a possible dietary supplement for animals after weaning.

## Introduction

Weaning is a period during which there is significant change in the magnitude and diversity of exposure to environmental antigens derived from food and potentially pathogenic organisms (Magne *et al*., 2006). During and shortly after weaning, the immature gastrointestinal tract (GIT) of animals must adapt to food composition and bacterial colonization, leading to mucosal surface stimulation from both bacterial and dietary antigens (Pohl *et al*., 2015; Wang *et al*., 2015a). As a result, animals at the weaning stage become more susceptible to infection, disorders and diarrhoea, resulting in post‐weaning maldigestion and malabsorption (Lallès *et al*., 2007). Developing a strategy to stimulate the intestinal growth and development of early‐weaned animals may help to optimize their performance and health during this critical stage.

Numerous studies have indicated that intestinal hormones, such as epidermal growth factor (EGF) and glucagon‐like peptide‐2 (GLP‐2), play a significant role in the decrease of weaning stress (Washizawa *et al*., 2004; Wang *et al*., 2015a). In recent years, remarkable advances based on studies of EGF and GLP‐2 in human and animal models (e.g. rat and piglet) have deepened our understanding regarding the influences of intestinal hormones on the structure and function of the GIT (Chen *et al*., 2002; Qi *et al*., 2015; Wang *et al*., 2015a; Wang *et al*., 2015b). In fact, the dramatically decreased intake of GLP‐2 may be a vital cause of decreased mucosal defence and reduced absorptive function (Xu *et al*., 2000; Cummins and Thompson, 2002). In addition to being a newly discovered hormone that is uniquely trophic for the GIT, GLP‐2 is also a specific growth factor that is used as a novel treatment modality in a variety of conditions with intestinal injury (Wallis *et al*., 2007; Connor *et al*., 2015). Until now, studies regarding the effects of GLP‐2 on experimental subjects, such as mice (Iakoubov *et al*., 2009), rats (Koopmann *et al*., 2008; Pedersen *et al*., 2015) and pigs (Qi *et al*., 2015) have shown similar results. Consequently, previous results have also indicated that an exogenous GLP‐2 supplement may be effectively taken up by animals. Additionally, the therapeutic potential of exogenous GLP‐2 as a meaningful method for intestinal development and infection during weaning has also been shown in paramount studies (Qi *et al*., 2014; Sigalet *et al*., 2014; Suri *et al*., 2014; Thymann *et al*., 2014a; Baldassano *et al*., 2016). However, the production and purification of recombinant GLP‐2 using conventional methods are expensive processes. Thus, a cost‐effective approach to highly efficiently express and deliver recombinant GLP‐2 to the GIT of animals after weaning in general is lacking.

*Saccharomyces cerevisiae* (*S. cerevisiae*) is a non‐invasive, non‐pathogenic and non‐colonizing eucaryote that is mainly used as a common tool for protein expression in research, industry and medicine (Romanos *et al*., 1992). *S. cerevisiae* is also metabolically active in all compartments of the GIT, making live delivery of recombinant functional proteins to the sites in the intestine possible (Wang *et al*., 2015a,b). The potential of *S. cerevisiae* to generate fully biologically active EGF or other growth factors has been shown in the recent studies. For example, Wang *et al*. (2015a) have constructed the recombinant EGF‐expressing *S. cerevisiae*, which can survive inside the GIT rather than outside, thus avoiding the risk of the genetically modified bacterium influencing the environment (Wang *et al*., 2015a). Herein, the current study will test the hypothesis that the diet of rats supplemented with the recombinant strain of GLP‐2‐expressing *S. cerevisiae* can stimulate intestinal crypt cells and enhance intestinal development or integrity, helping to improve the intestinal health and growth of weaned animals.

## Materials and methods

### Cloning of the pYES2‐GLP‐2 expression construct

According to the gene sequence of GLP‐2 (NP‐999489), the complete GLP‐2 gene was synthesized by Invitrogen Co (Invitrogen, Shanghai, China). The plasmid pMD19‐GLP‐2 was linearized with KpnI and XhoI. Subsequently, the purified GLP‐2 insert was cloned into multiple cloning sites of the 5,962 bp expression vector pYES2/CT (Invitrogen, CA, USA). The recombinant construct was designated the plasmid of pYES2‐GLP‐2 that contained a *yeast* GAL1 promoter, a URA3 gene, a versatile multiple cloning site and an ampicillin resistance gene. Thereafter, the plasmid of pYES2‐GLP‐2 was transformed into *S. cerevisiae* (INVSc1) (Invitrogen, USA) using the chemical method. The transformant was designated GLP2‐SC and expressed the GLP‐2 protein. PCR identification of the GLP2‐SC strain was performed using the primer pair GLP2‐F (5′‐CGGGATCCAAAAAAATGCATGGTGATGGTTCT‐3′) and the reverse primers GLP2‐R (5′‐CCCTCGAGTTATTCAGTAACTTTAGT‐3′). The PCR programme was as follows: 94°C (5 min), followed by 30 cycles of 94°C (45 s), 60°C (30 s) and 72°C (30 s), with a final extension at 72°C (10 min). The original pYES2/CT plasmid (without the GLP‐2 DNA insert) was transformed into *S. cerevisiae* as a control, which was designated EV‐SC in the current research.

### Growth and fermentation of the recombinant GLP2‐S.C strain

Frozen inoculum stocks of the GLP2‐SC strain were stored in 20% glycerol (vol/vol) at −80°C. The glycerol stocks of the GLP2‐S.C strain were streaked on SC‐U agar plates (with 1 *μg*/mL ampicillin and 2% D‐raffinose) and cultured at 30°C for approximately 24 h. A single colony was inoculated into 20 mL of fresh SC‐U medium (with 1 *μ*g/mL ampicillin and 2% D‐raffinose) and then was incubated at 30°C overnight. The growth of the GLP2‐S.C strain was also detected at different time points from 0 to 48 h. In addition, the concentration of the GLP2‐S.C strain was assayed by the optical density at 600 nm (OD_600_) using spectrophotometry, and its pH value was simultaneously measured with a digital pH meter (Fisher Scientific, Pittsburgh, PA, USA). At each time point, 10 mL of culture was centrifuged at 5000 r.p.m. for 10 min at 4°C, and then the cell pellets of the GLP2‐S.C strain were stored at −80°C for subsequent analysis.

### Western blotting and quantitative analysis of the recombinant GLP‐2‐expressing *S. cerevisiae*

The cell pellets from the GLP2‐SC strain were thawed, and the cell pellets were lysed with the Yeast Total Protein Extraction Kit (Zoman Biotechnology, Beijing, China), after which the cell lysates were filtered with Centrifugal Filter Devices (Millipore, Boston, MA, USA). The recombinant GLP‐2 protein was separated by 16.5% tricine‐sodium dodecyl sulfate‐polyacrylamide gel electrophoresis (Tricine‐SDS‐PAGE) and then was transferred to polyvinylidene fluoride membranes at 4°C for 90 min. The blots were blocked at 30°C for 60 min in phosphate‐buffered saline (PBS) containing 5% skimmed milk and then were incubated with the primary anti‐GLP‐2 antibody (1:300 dilution; Keshun Biological Technology, Shanghai, China) overnight at 4°C. The membranes were washed in PBS‐0.2% Tween 20 and were subsequently blotted in an anti‐rat IgG horseradish peroxidase‐linked antibody (1:2,000 dilution; ComWin Biotech, Beijing, China) at 30°C for 60 min. After washing, the DAB Western Blotting Detection System kit (ComWin Biotech) was used to visualize the target protein (GLP‐2) band.

### In vitro rat enterocyte proliferation assay

The rat enterocytes (provided by Southwest University for Nationalities) were adhered to the bottom of 25 cm^2^ cell culture flasks (Corning, New York, NY, USA) and then were overlaid with 5 mL of Dulbecco's modified Eagle's medium (DMEM) supplemented with 10% fetal bovine serum (FBS). The culture was maintained at 37°C in an atmosphere of 10% CO_2_/air. The culture medium was changed daily. The primary cell monolayer was trypsinized when confluent and was split 1:2 during subculturing. The cells at passage 4 were used for the proliferation assessment, which was conducted approximately 4 weeks after the initial tissue harvesting. The cells were then seeded into the cell culture flasks at an initial cell density of approximately 0.35 × 10^5^ cells. The culture medium was aspirated by vacuum, and the attached cells were washed twice with 1× sterile PBS.

The rat enterocytes were incubated in DMEM for 24 h to reach serum starvation. Next, the cells were divided into four groups, and each group was subjected to the following treatments: recombinant human GLP‐2 (rh‐GLP2; 20 ng) (Phoenix Pharmaceuticals, Los Angeles, USA); the GLP2‐SC cell lysate (GLP‐2; 20 ng); the EV‐SC cell lysate (the same volume); and the control group (1× PBS, the same volume) respectively. After 24 h, the cells were trypsinized and then quantified in a haemocytometer chamber. The cell counting was performed in a blinded manner, and the results of cell counting represented the mean data that were collected by three individuals who were not aware of the experimental treatments in the current research. Finally, the cell counting for each group represented the means ± SEM from three experiments.

### Animal experiments

#### Diet supplemented with the recombinant GLP2‐S.C strain

The animal use procedures were performed in accordance with the guidelines of the China Small Animal Protection Association, and all studies were approved by the Animal Care Committee in West China Hospital of Sichuan University.

Weaned 21‐day‐old rats were assigned randomly to one of the following four groups: control group (12 rats), EV‐SC group (12 rats), GLP2‐SC group (12 rats), or rh‐GLP2 group (12 rats). All of the rats in the current study were housed in a temperature‐controlled environment with a cycle of 12‐h light/12‐h dark and were provided free access to water. The basic diet (Table S1) of the rats was formulated without any in‐feed antibiotics based on a previous report (Reeves *et al*., 1993). For each feeding, the rats in the EV‐SC and GLP2‐SC groups were supplemented daily with approximately 450 *μ*L of fresh bacterial culture of the EV‐SC and GLP2‐SC strains, respectively, for 14 continuous days. The rats in the rh‐GLP2 group also ingested approximately 60 *μ*g/kg of recombinant human GLP‐2 daily in the same volume of sterile water (450 *μ*L). The control group was only provided sterile water (the same volume). Thus, all of the 48 rats were used in the current study, and a maximum of three rats were housed in each cage within the same treatment group. The initial body weight and food intake were recorded daily, and signs of diarrhoea, sickness or abnormal behaviour were also monitored throughout the experimental period. On day 15, all of the rats were subsequently sacrificed with sodium pentobarbital (50.00 mg/kg/body weight) via intravenous injections to sample the jejunum.

#### Assessment of the morphology of the jejunum

The jejunum samples were prepared in accordance with a previously described procedure (Wang *et al*., 2015a). Approximately 1.5–2.0 cm jejunal segments were rinsed with 1× PBS and then were fixed in 10% formalin to assess the jejunal morphology and crypt cell. Thereafter, the samples were subjected to morphological examination using the haematoxylin and eosin (H&E) staining method in Lilai biological laboratory. Briefly, the previously fixed samples were embedded in paraffin, and then approximately 4–5 μm jejunal sections were mounted on poly‐Lys‐coated slides, deparaffinized and rehydrated. The slide from each sample was stained with H&E to measure the morphology of the jejunum. Finally, light microscopy analyses at both ×10 and ×40 magnification were also performed on the paraffin‐embedded jejunal sections with H&E staining to examine the mucosa morphology of the jejunum after the respective treatments. Approximately, 10 selected villi and crypts from each sample were marked by a measuring scale using Motic Images Advanced 3.2 (Motic, Shanghai, China) and then were measured in accordance with the size ratio of the measuring scale.

#### Immunohistochemistry analysis of proliferating cell nuclear antigen

The protocol of immunohistochemistry was conducted in accordance with a previous published method (Cheung *et al*., 2009). Briefly, the slides containing paraffin‐embedded tissues were first deparaffinized by 100% xylene and rehydrated by decreasing the percentages of the ethanol. Subsequently, the slides were then incubated in a bath (containing 10 mmol/L sodium citrate) at 90°C for 60 min for antigen retrieval and then were transferred to a bath of 0.20% sodium borohydride to block the endogenous peroxides.

After washing three times with 1× PBS, the slides were blocked in blocking buffer that contained 0.025% Triton X‐100, 2% FBS and 5% BSA at 25°C for 60 min. The slides were further incubated in monoclonal‐proliferating cell nuclear antigen (PCNA) antibody (1:500 dilution; BIOSS, Beijing, China) overnight at 4°C in a humidified chamber. The slides were then incubated with the biotin‐conjugated goat anti‐pig immunoglobulins (1:250 dilution; BIOSS) at 25°C for 90 min when the primary antibody was rinsed off. Thereafter, the slides were further treated with horseradish peroxidase‐conjugated streptavidin (ComWin Biotech) at 25°C for 30 min and were then reacted with 1 mL of 3,3‐diaminobenzidine tetrahydrochloride (ComWin Biotech) to visualize the antigenic structures. Finally, the slides were counterstained with Harris Haematoxylin, mounted onto coverslips using the liquid mounting medium and assessed under bright‐field light microscopy at 100× magnification.

#### Determination of the digestive enzyme activities of the jejunal mucosa

The activities of digestive enzymes, including alkaline phosphatase (ALP), creatine kinase (CK) and lactate dehydrogenase (LDH), in the jejunal mucosa were assayed using enzyme activity detection kits (NanJingJianCheng Bioengineering Institute, Nanjing, China) in accordance with the instructions respectively.

### Statistical analysis

The data of the growth, morphology, digestive enzyme activities and crypt cells were evaluated; each pen was considered an experimental unit in the current research. According to the PROC ANOVA procedure performed using SAS software (Version 9.0, Shanghai, China), the results were analysed using the multi‐way ANOVA with different treatments as the single factor. Once the multi‐way ANOVA results were significant, Duncan's multiple‐range test was further performed for multiple comparisons. A *P* < 0.05 was considered to be statistically significant in the current research.

## Results

### Generation of recombinant GLP‐2‐expressing *S. cerevisiae*

**Figure 1:**
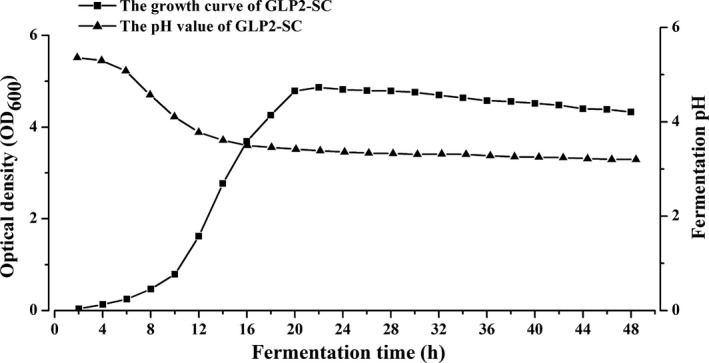
The growth of recombinant *S. cerevisiae* (GLP2‐SC) during the culture period. The OD_600_ showed that the GLP2‐SC strain achieved similar growth characteristics during 48‐h fermentation monitoring. The pH values of the culture remained at 3.39–5.35 from 0 to 48 h.

**Figure 2:**
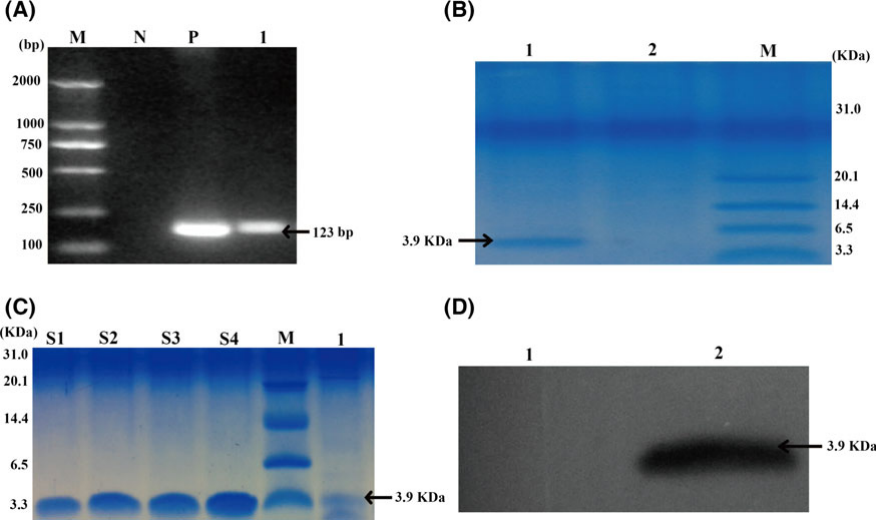
Identification of GLP‐2‐expressing recombinant *S. cerevisiae* (GLP2‐SC). (A) The PCR profile of recombinant *S. cerevisiae* (GLP2‐SC). Lane M: D_2000_ DNA marker (100–2000 bp); Lane N: negative control (ddH_2_O); Lane P: positive control (pYES2‐GLP‐2 from *E. coli* transformant); Lane 1: colonies of pYES2‐GLP‐2 from *S. cerevisiae*. (B) Tricine‐SDS‐PAGE analysis of GLP‐2 protein generated by recombinant *S. cerevisiae* (GLP2‐SC). Lane M: protein marker (3.3–31 kDa); Lane 1: cell lysates from *S. cerevisiae* transformed with GLP2 (GLP‐SC strain); Lane 2: cell lysates from *S. cerevisiae* transformed with the empty vector backbone (the EV‐SC strain). Recombinant GLP‐2 protein was indicated with the arrowheads at approximately 3.9 kDa. (C) Comparison of the intensities of recombinant human GLP‐2 protein (rh‐GLP2) with GLP‐2 produced by recombinant *S. cerevisiae* (GLP2‐SC) by tricine‐SDS‐PAGE analysis. Known concentrations of rh‐GLP2 were loaded on the same tricine‐SDS‐PAGE gel to estimate the approximate production of GLP‐2 protein by recombinant *S. cerevisiae* (GLP2‐SC). Lane M: the protein marker (3.3–31 kDa); Lane S1‐S4: rh‐GLP2 at 20.00, 25.00, 30.00 and 35.00 ng respectively. Lane 1: approximately 10 *μ*L of cell lysates from recombinant *S. cerevisiae* (GLP2‐SC) was loaded per well. (D) Western blotting analysis of GLP‐2 protein produced by recombinant *S. cerevisiae* (GLP2‐SC). Lane 1: cell lysates from *S. cerevisiae* transformed with the empty vector backbone (the EV‐SC strain); Lane 2: cell lysates from *S. cerevisiae* transformed with GLP‐2 (the GLP‐SC strain); recombinant GLP‐2 protein is indicated with an arrowhead.

A fragment of the GLP‐2 gene was inserted into the expression vector pYES2/CT to generate the recombinant *S. cerevisiae* GLP2‐S.C, which was then identified by PCR amplification, as shown in Fig. 2A. In addition, the growth curve and pH value measured during a 48‐h fermentation period were shown in Fig. 1. The maximal growth of GLP2‐S.C appeared at 22 h with an OD_600_ of 4.80. The initial pH of the culture liquid was 5.35, which decreased to 3.39 at 22 h, indicating active fermentation. The decline in the pH to <5 should activate the expression of GLP‐2 via the strong *GAL1* promoter.

The recombinant GLP2‐S.C strain could produce GLP‐2 protein (≈3.9 kDa), which was detected using tricine‐SDS‐PAGE analysis (Fig. 2B). In addition, as shown in Fig. 2D, Western blotting analysis was further performed to analyse the GLP‐2 protein using a specific antibody against GLP‐2 protein. The results showed that GLP‐2 protein was generated and secreted by the recombinant GLP2‐S.C strain. By comparing the intensities of the bands derived from the commercial recombinant human GLP‐2 protein standards with those of the bands from these samples, we determined that ≈1.35 mg/L GLP‐2 was present at the culture of the recombinant GLP2‐S.C.

### The GLP‐2 protein produced by recombinant *S. cerevisiae* (GLP2‐SC) is functional in vitro

To clarify whether the GLP‐2 protein generated and secreted from the GLP2‐S.C strain was indeed functional, an in vitro assay of cell proliferation was performed in the current study. The rat enterocytes were cultured in the absence or presence of GLP‐2 protein from GLP2‐S.C cultures for 24 h. The cells were trypsinized and then were enumerated using a haemocytometer.

**Figure 3:**
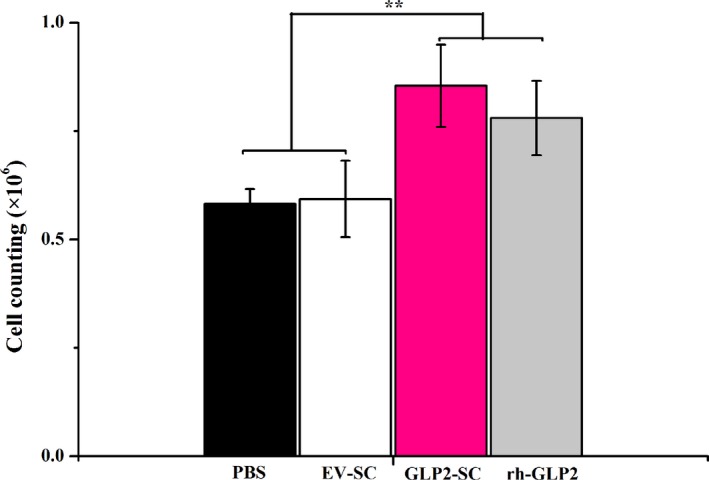
GLP‐2 secreted by recombinant *S. cerevisiae* (GLP2‐SC) is functional in vitro. Rat enterocytes were treated with 1× PBS, cell lysates from *S. cerevisiae* transformed with the empty vector backbone (the EV‐SC strain) and GLP2 (the GLP‐SC strain) and recombinant human GLP‐2 (rh‐GLP2) respectively. The results were presented as the means ± SEM of three experiments. The symbol ‘**’ indicates statistically significant differences, *P* < 0.01.

As revealed in Fig. 3, the proliferation of rat enterocytes was stimulated significantly by GLP‐2 protein from the GLP2‐S.C culture (0.85 × 10^6^ ± 0.10 cells) compared with the control group (1× PBS) (0.58 × 10^6^ ± 0.03 cells; *P* < 0.01) or empty vector‐bearing *S. cerevisia*e (EV‐SC) (0.59 × 10^6^ ± 0.15 cells; *P* < 0.01), whereas these results were comparable to those obtained in the positive control group (rh‐GLP2) (0.78 × 10^6^ ± 0.09; *P* > 0.05).

### Effects of the diet of weaned rats supplemented with the live GLP2‐S.C strain on growth

The biological activities of GLP2‐S.C were further assessed in vivo by feeding the diet supplemented with the live GLP2‐SC strain to weaned rats. During the experimental period, no abnormal behaviour or diarrhoea was observed.

**Figure 4:**
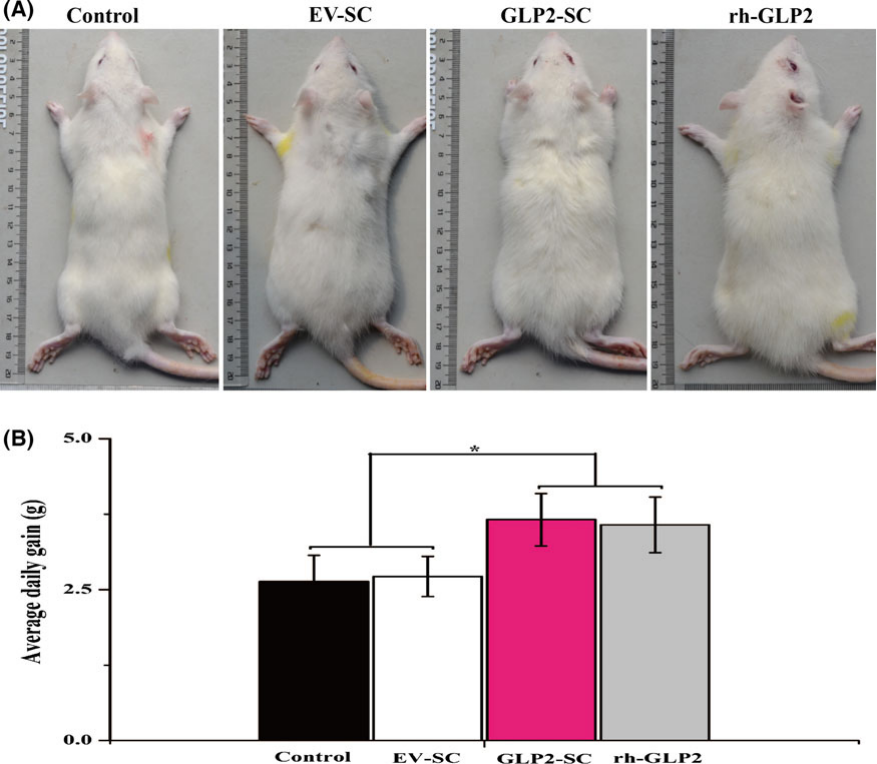
Effects of the diet of weaned rats supplemented with the live GLP2‐S.C strain on the growth in vivo. (A) Representative photograph of body size differences of the rats receiving the different treatments on day 15. (B) Comparison of the average daily gain (ADG) after treatment for 15 days with the control group (the sterile water; 12 rats), EV‐SC group (*S. cerevisiae* transformed with the empty vector backbone; 12 rats), GLP2‐SC group (*S. cerevisiae* transformed with GLP‐2; 12 rats) and rh‐GLP2 group (recombinant human GLP‐2; 12 rats). The symbol ‘*’ indicates statistically significant differences, *P* < 0.05.

By day 15, no obvious differences were observed in the food intake among the groups tested (*P* > 0.05). The ADG of rats fed GLP2‐S.C (3.66 ± 0.73 g) was significantly higher than that in the control group (2.63 ± 0.74 g) or SC(EV) group (2.72 ± 0.73 g; *P* < 0.05) but was comparable to that in the rh‐GLP2 group (3.57 ± 0.70 g; *P* > 0.05; Fig. 4B). Additionally, the rats fed the GLP2‐S.C strain appeared generally larger than the rats fed the control or EV‐SC (Fig. 4A).

### Recombinant GLP‐2‐expressing *S. cerevisiae* stimulates the intestinal development of weaned rats

#### The effects of the diet of weaned rats supplemented with live GLP2‐S.C strain on the morphology of the jejunum

**Figure 5:**
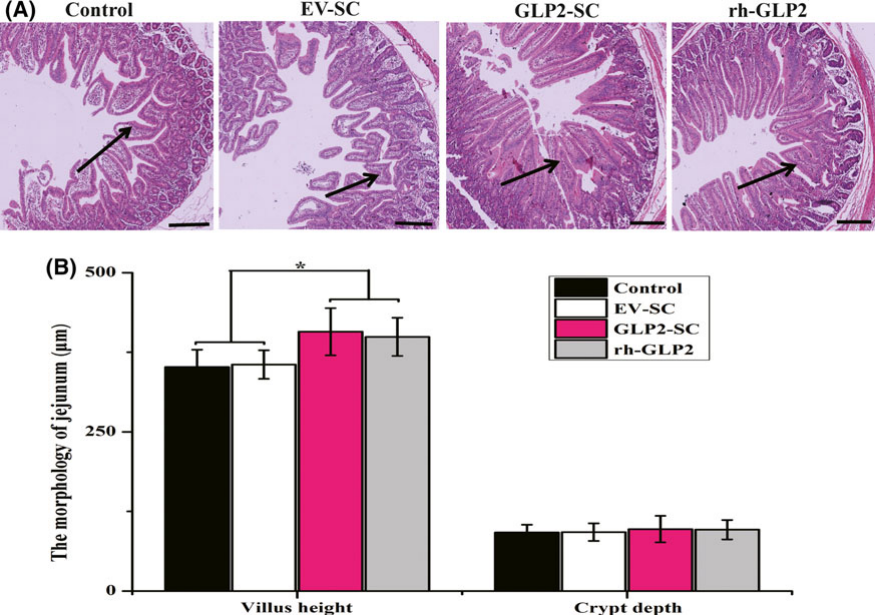
Effects of the diet of weaned rats supplemented with the live GLP2‐S.C strain on the morphology of the jejunum in vivo. (A) Representative light micrograph of a cross‐section taken at the jejunum from the control group (the sterile water), EV‐SC group (*S. cerevisiae* transformed with the empty vector backbone), GLP2‐SC group (*S. cerevisiae* transformed with GLP‐2) and rh‐GLP2 group (recombinant human GLP‐2). Images were taken at 40× magnification using the light microscope, and the scale bar is equivalent to 100 *μ*m. (B) Comparison of the villus height and crypt depth in the jejunum among all of the groups, including the control, EC‐SC, GLP2‐SC and rh‐GLP2 groups. The symbol ‘*’ indicates statistically significant differences, *P* < 0.05.

The morphology of the jejunum was assessed by measuring the villous height and crypt depth simultaneously. As indicated in Fig. 5A, the villi in the jejunum obtained from the rats fed GLP2‐S.C were longer than those from the control or EV‐SC groups but were comparable to those in the rh‐GLP2 group. Furthermore, the differences among them were confirmed by a quantification study using image analysis software (Motic Images Advanced 3.2). The average villous height in the jejunum was also maximal in the GLP2‐S.C group (*P* < 0.05; Fig. 5B). However, in terms of the crypt depth in the jejunum (Fig. 5B), there was no significant difference among all of the groups tested (*P* > 0.05).

#### Recombinant GLP‐2‐expressing *S. cerevisiae* stimulated the proliferation of crypt cells in the jejunum

**Figure 6:**
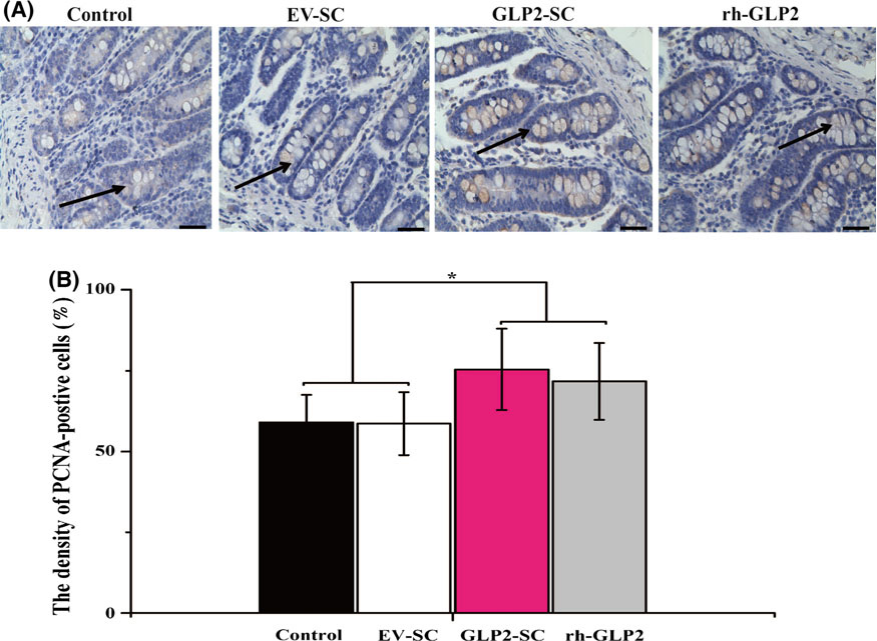
Effects of the diet of weaned rats supplemented with the live GLP2‐S.C strain on the proliferation of crypt cells in the jejunum in vivo. (A) Representative images of the immunohistochemical detection of PCNA taken at the jejunum from the control group (the sterile water), EV‐SC group (*S. cerevisiae* transformed with the empty vector backbone), GLP2‐SC group (*S. cerevisiae* transformed with GLP‐2) and rh‐GLP2 group (recombinant human GLP‐2). The images were taken at 100× magnification; the scale bar is equivalent to 10 *μ*m. (B) Density of PCNA‐positive cells taken at the jejunum from the control, EC‐SC, GLP2‐SC and rh‐GLP2 groups respectively. The results were expressed as the means ± SEM of six experiments. The symbol ‘*’ indicates statistically significant differences, *P* < 0.05.

To monitor the status of enterocyte proliferation in the different treatments, immunohistochemical analyses were performed on the paraffin‐embedded jejunal tissue sections using an antibody against PCNA. As revealed in Fig. 6A, most PCNA‐positive cells were localized in the crypt area, whereas some proliferating cells had migrated along the villi (arrowheads). When the crypt cells were examined at a higher magnification, approximately 75% and 72% of the nuclei in the GLP2‐S.C group and the positive control group (rh‐GLP2) were positive for PCNA, respectively, compared with approximately 58% and 60% in the control group and EV‐SC group respectively (Fig. 6B).

#### Recombinant GLP‐2‐expressing *S. cerevisiae* enhances the activities of digestive enzymes in the jejunum

**Figure 7:**
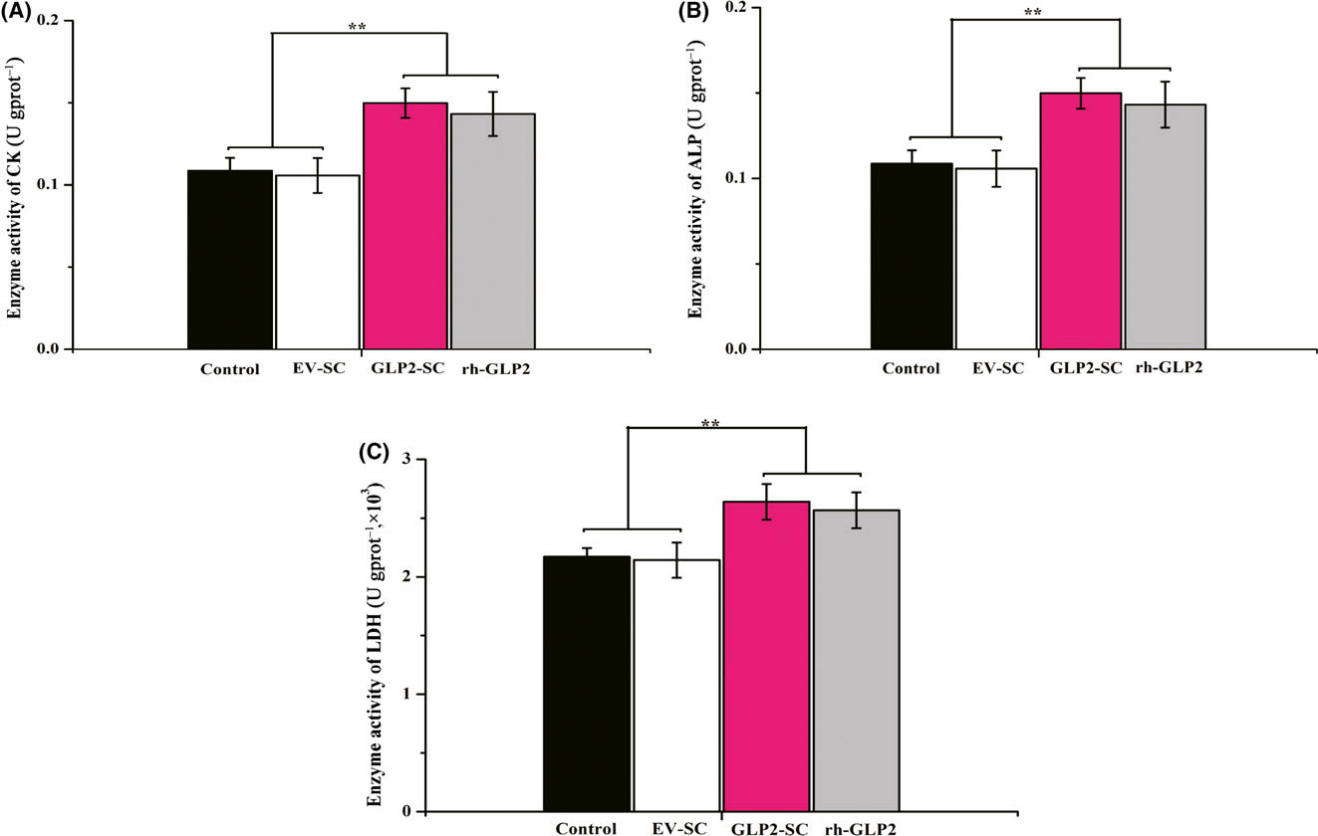
Effects of the diet of weaned rats supplemented with the live GLP2‐S.C strain on the digestive enzyme activities of the jejunum in vivo. (A) Activities of creatine kinase (CK) in the jejunum from the control group (the sterile water), EV‐SC group (*S. cerevisiae* transformed with the empty vector backbone), GLP2‐SC group (*S. cerevisiae* transformed with GLP‐2) and rh‐GLP2 group (recombinant human GLP‐2) respectively. The symbol ‘**’ indicates statistically significant differences, *P* < 0.01. (B) Activities of alkaline phosphatase (ALP) in the jejunum from the control, EC‐SC, GLP2‐SC and rh‐GLP2 groups respectively. The symbol ‘**’ indicates statistically significant differences, *P* < 0.01. (C) Activities of lactate dehydrogenase (LDH) in the jejunum from the control, EC‐SC, GLP2‐SC and rh‐GLP2 groups respectively. The symbol ‘**’ indicates statistically significant differences, *P* < 0.01.

As revealed in Fig. 7, by day 15, the activities of digestive enzymes encompassing CK, ALP and LDH in the jejunum were significantly higher in the GLP2‐S.C group than in the control and EV‐SC groups *(P* < 0.01) but were comparable to those in the rh‐GLP2 group (*P* > 0.05).

## Discussion

In the small intestine, a pro‐glucagon gene in the enteroendocrine *L* cells undergoes specific post‐translational cleaving, thereby releasing the GLP‐2 protein (Hansen *et al*., 2007; Wallis *et al*., 2007; Hua *et al*., 2013). GLP‐2 motivates multiple effects mainly associated with the regulation of gastrointestinal development and function, such as stimulating intestinal mucosal growth, enhancing nutrient absorption, reducing intestinal permeability, inhibiting gastric emptying and secreting gastric acid (Au *et al*., 2002; Yu *et al*., 2014). However, during weaning, diarrhoea is completely absent in highly sanitary conditions, and the native GLP‐2 in the small intestine fails to affect the structure and function of the intestine (Yu *et al*., 2014). Thus, the dramatically decreased intake of GLP‐2 after weaning may be a crucial cause of the reduction of absorptive functions and degradation of mucosal defences. Interestingly, the GLP‐2 receptor, which is predominantly expressed in the jejunum, distinctly regulates the functions of GLP‐2 in the GIT (Yusta *et al*., 1999, 2012). Growing evidence has shown that exogenous GLP‐2 might be effectively absorbed by young animals via receptor activation (Rowland and Brubaker, 2008; Leen *et al*., 2011; Angelone *et al*., 2012; Hua *et al*., 2013).

In the current study, we have indicated that a diet supplemented with recombinant GLP‐2‐expressing *S. cerevisiae* could stimulate intestinal development to increase the ADG of weaned rats. Our results also supported previous reports regarding the role of exogenous GLP‐2 in other animals. For example, one study reported a 200% increase in the activities of sucrase and maltase, a 50% increase in the epithelial volume of the intestine, increased DNA and protein content and increased synthesis rate of total protein in short bowel syndrome (SBS)+GLP‐2 versus SBS pigs (Vegge *et al*., 2013). Qi *et al*. (2014) had also reported that PEG‐pGLP‐2 infusion alleviated the severity of intestinal injury in weaned piglets by reducing the secretion of inflammatory cytokines and caspase‐3 activity and increasing the villus height/crypt depth ratio (Qi *et al*., 2014). In addition, in weaned piglets exposed to *Escherichia coli* lipopolysaccharide, Qi *et al*. (2015) further indicated that GLP‐2 reduced the expression of intestinal pro‐inflammatory cytokines, such as TNF‐alpha, IL‐8 and IL‐10, via stimulating the release of the GLP‐2R mediator (Qi *et al*., 2015). In another study, chronic treatment of weaning pigs with GLP‐2 at a dose of 40 *μ*g/kg/day improved the overall villus height and crypt depth due to an increase in crypt cell proliferation rates and a decrease in apoptosis (Sigalet *et al*., 2014). Additionally, GLP‐2 significantly stimulated brush border enzymes and goblet cell density to improve the function of the GIT in weaned piglets (Thymann *et al*., 2014a). As the supplement of intestinal hormones, GLP‐2 improved the clinical, histological and morphological outcomes of the intestinal adaptation in the distal‐intestinal resection model of SBS (Suri *et al*., 2014). Collectively, these data indicate that the administration of GLP‐2 indeed affected gut function and diarrhoea sensitivity during and shortly after weaning.

In recent decades, the fundamentals of small intestinal growth and the importance of the crypt cells in maintaining the homeostasis of the GIT were demonstrated in numerous studies (Koopmann *et al*., 2008; Rowland and Brubaker, 2008; de Heuvel *et al*., 2012; Yusta *et al*., 2012; Vegge *et al*., 2013; Thymann *et al*., 2014a). Healthy intestinal morphology was determined by the equilibrium of the epithelial cell turnover, and decreased cell proliferation and increased apoptosis might be the primary mechanisms responsible for intestinal mucosal injury (Sigalet *et al*., 2006). As an intestinal growth factor, GLP‐2 could stimulate the regulation of nutrient uptake in the GIT and maintain a normal morphology in the gut epithelium (Connor *et al*., 2015). Thus, GLP‐2 supplementation in mice or rats reduces enterocyte apoptosis and, to a lesser extent, increases enterocyte proliferation (Tsai *et al*., 1997; Burrin *et al*., 2000; Ghatei *et al*., 2001). In the current study, recombinant GLP‐2 protein stimulated enterocyte proliferation in vivo or in vitro to enhance the intestinal growth of weaned rats. The current results were similar to those in previous reports that GLP‐2 stimulated the proliferation of crypt cells (Rowland and Brubaker, 2008) and intestinal subepithelial fibroblasts (Leen *et al*., 2011). The trophic effect of GLP‐2 supplementation was also a result of the inhibitory effects on apoptosis in crypt cells. A growing number of studies in rodents and pig models have indicated that GLP‐2 decreases the ratio of apoptotic cells in both the crypt and villus tips of the intestine (Burrin *et al*., 2000; Shin *et al*., 2005; Sigalet *et al*., 2007). This action mechanism of GLP‐2 involved the upregulation of Bcl‐2 and suppression of caspase‐3 activity, followed by an increase in the cellular inhibitor of apoptosis and X‐linked inhibitor of apoptosis (Burrin, *et al*., 2007), which suppress the activities of pro‐apoptotic proteins, including caspases‐3, caspase‐7 and caspase‐9 (Nachmias *et al*., 2004). Within the crypt, these effects of GLP‐2 likely contribute to the maintenance of the crypt cell population via the balance between apoptosis and proliferation (Rowland and Brubaker, 2008). As with the current hypothesis regarding the roles of GLP‐2 in the GIT, GLP‐2 stimulated intestinal growth by inhibiting apoptosis and increasing cell proliferation in the crypt.

Additionally, the diets supplemented with GLP‐2 also elevated the enzymatic activities of ALP, CK and LDH in the intestinal mucosa of weaned rats in the present work. These brush border enzymes were the markers of villus maturation, and their activities served as a marker of small intestine injury in several studies (Lackeyram *et al*., 2010; Pi, *et al*., 2014, Wang *et al*., 2015a,b, 2016). The results in the current study are consistent with the data published by other studies that GLP‐2 increased the activities of brush border enzymes in mice, rats and neonatal piglets (Petersen *et al*., 2002; Jia *et al*., 2009; Qi *et al*., 2014; Sigalet *et al*., 2014). Nevertheless, other reports demonstrated that the activities of these digestive enzymes were not influenced by GLP‐2 supplementation. Petersen *et al*. (2001) showed that the activities of digestive enzymes were unaffected by GLP‐2 in late‐gestation fetal and parenterally fed neonatal piglets when GLP‐2 (12.5 nmol/kg) was administered twice daily for 6 days (Petersen *et al*., 2001). The newborn total parenteral nutrition‐fed piglets were subjected to daily single administration of GLP‐2 for 7 days; as a result, small bowel growth was increased, and a minimal effect was elicited on digestive enzyme expression or nutrient absorption (Thymann *et al*., 2014b). Collectively, these different effects of GLP‐2 on the small intestinal brush border function might be affected by multiple factors, such as gestational age at birth (Petersen *et al*., 2002), units of enzyme activity (Brubaker *et al*., 1997) or GLP‐2 analogues (Thymann *et al*., 2014a). In the current study, these differences regarding brush border enzymes, including ALP, CK and LDH, might be significant only in rats that develop weaning stress.

Considering the commercially available recombinant GLP‐2 as a standard, it was estimated that the concentration of GLP‐2 in the culture cell lysates in a 48‐h culture was ≈1.35 mg/mL. The actual amount of GLP‐2 delivered was ≈600 *μ*g/day with the feeding of ≈450 *μ*L GLP2‐SC cells per day, which amounted to ≈60 *μ*g/kg daily. Thus, this dosage of GLP‐2 protein was equivalent to that of the effective doses of other growth‐stimulating factors contributing to intestinal development and growth (Cheung *et al*., 2009; Wang *et al*., 2015b). In addition, repeated determinations of the OD_600_ revealed that the growth peak of recombinant *S. cerevisiae* (GLP2‐SC) was at 22 h. The current results further supported previous reports that the growth of recombinant *S. cerevisiae* peaked at approximately 20 h (Wang *et al*., 2015b). Subsequently, we also established fermentation parameters for the target protein at 30°C and a pH of 3.3. This finding was different from the data reported by previous studies (Valdés *et al*., 2009), which proposed that the optimal fermentation parameters for human EGF were a temperature of 25–30°C and a pH of 5.0 to 6.5. Therefore, it is necessary to further optimize the temperature, time, medium and pH conditions for GLP‐2 protein expression and control proteolysis to achieve highly efficient and large‐scale GLP‐2 production via zymotechnics in future research.

In conclusion, the now available research demonstrated that GLP2‐SC promoted the intestinal development and growth of weaned rats. The results in the current study also indicated that formulations containing GLP‐2 combined with probiotics could prevent weaning stress and the related reduction in growth performance in other early‐weaned animals or children during their transition from the weaning stage.

## Conflicts of interest

None of the authors of this paper has a financial or personal relationship with other people or organizations that could inappropriately influence or bias the content of the paper.

## Supporting information


**Table S1.** Formula and nutrient content of the basic diet.
