# Supplementary material for: Generation of glucagon‐like peptide‐2‐expressing Saccharomyces cerevisiae and its improvement of the intestinal health of weaned rats
Source: Microb Biotechnol. 2016 Sep 19;9(6):846–57. doi: 10.1111/1751-7915.12412 (PMC5072200; doi:10.1111/1751-7915.12412)
Supplement: Supplementary file 1 — Table S1. Formula and nutrient content of the basic diet. [file MBT2-9-846-s001.docx]

**Table S1. Formula and nutrient content of the basic diet.**

| Ingredients | Content |
| --- | --- |
| Corn meal, % | 51.74 |
| Soybean meal, % | 22.10 |
| Whey powder, % | 5.00 |
| Wheat flour, % | 9.00 |
| Wheat bran, % | 5.00 |
| NaCl, % | 0.30 |
| CaHPO_4_, % | 1.15 |
| CaCO_3_, % | 1.65 |
| Soybean oil, % | 3.00 |
| Sucrose, % | 0.10 |
| Mineral premix^1^, % | 0.06 |
| Vitamin premix^2^, % | 0.02 |
| Choline chloride, % | 0.20 |
| Methionine, % | 0.20 |
| Lysine, % | 0.30 |
| L-Cysteine, % | 0.18 |
| Total | 100.00 |
| Nutrition level^3^ |  |
| Metabolizable Energy, MJ/kg | 3.51 |
| Crude protein, % | 18.65 |
| Ca, % | 1.01 |
| P, % | 0.59 |

Note：**^1^**Mineral premix provides the following per kg of diet: FeSO_4_.7H_2_O, 251.41 mg; CuSO_4_.5H_2_O, 23.81 mg; MnSO_4_. H_2_O, 155.37 mg; ZnSO_4_.7H_2_O, 132.59 mg; Na_2_SeO_3_, 0.22 mg; KI, 0.40 mg.

**^2^**Vitamin premix provides the following per kg of diet: Vitamin A, 4 000 IU; Vitamin D_3_, 1000 IU; Vitamin E, 50 IU; Vitamin B_1_, 6.00 mg; Vitamin B_2_, 6.00 mg; Vitamin B_3_, 30.00 mg; Vitamin B_5_, 16.00 mg; Vitamin B_6_, 7.00 mg; Vitamin B_11_, 2.00 mg; Vitamin B_12_, 0.01 mg; Vitamin H, 0.20 mg; Vitamin K_3_, 2.00 mg.

**^3^**Nutrition level being from calculated values.
